# *Homalium glandulosum* (Salicaceae), a new species from Vu Quang National Park, North Central Vietnam

**DOI:** 10.3897/phytokeys.58.6816

**Published:** 2016-01-12

**Authors:** Shuichiro Tagane, Viet Hung Nguyen, Nguyen Van Ngoc, Hoang Thanh Son, Hironori Toyama, Chen-Jui Yang, Tetsukazu Yahara

**Affiliations:** 1 Center for Asian Conservation Ecology, Kyushu University, 744 Motooka, Fukuoka, 819-0395, Japan; 2 Vu Quang National Park, Ha Tinh, Vietnam; 3 Department of Biology, Dalat University, 01 – Phu Dong Thien Vuong, Dalat, Vietnam; 4 Silviculture Research Institute, Vietnamese Academy of Forest Sciences, Ha Noi, 10999, Vietnam; 5 Institute of Ecology and Evolutionary Biology, National Taiwan University, Taipei, Taiwan

**Keywords:** Homalium, new species, Salicaceae, taxonomy, Vietnam, Vu Quang National Park

## Abstract

*Homalium glandulosum* Tagane & V. H. Nguyen, from Vu Quang National Park in northern Vietnam, is newly described. This species is characterized by distinct glands, often stalked, at the base of the lamina and along the margin of the stipules and bracteoles. Illustrations, DNA barcodes of the two regions of *rbcL* and *matK*, and a key to the species of *Homalium* in Vietnam are also provided.

## Introduction

*Homalium* Jacq., with more than 150 species, is a genus of woody plants widely distributed in the tropics of the world, with the center of diversity in Southeast Asia and Madagascar (Sleumer 1954, 1973, Applequist 2013). The genus is characterized by bisexual flowers with free petals and sepals, obconical calyx tube adnate to ovary in the lower part (i.e., semi-inferior ovary) and disk glands. It was previously classified in Flacourtiaceae (e.g. Sleumer 1954, Lescot 1970, Sleumer 1985, Yang and Zmarzty 2007) or Homaliaceae (Gagnepain 1921), but Chase et al. (2002) included it in Salicaceae *sensu lato* based on phylogenetic analyses of plastid *rbcL* DNA data.

In Vietnam, 11 species of *Homalium* have been known: *Homalium caryophyllaceum* (Zoll. & Moritz) Benth., *Homalium ceylanicum* (Gardner) Benth. (synonym, *Homalium balansae* Gagnep., *Homalium hainanense* Gagnep.), *Homalium cochinchinense* (Lour.) Druce (synonym, *Homalium digynum* Gagnep., *Homalium fagifolium* (Lindl.) Benth.), *Homalium dasyanthum* (Turcz.) W. Theob. (synonym, *Homalium griffithianum* Kurz), *Homalium dictyoneurum* (Hance) Warb., *Homalium grandiflorum* Benth., *Homalium mollissimum* Merr., *Homalium myriandrum* Merr., *Homalium petelotii* Merr., *Homalium phanerophlebium* F. C. How & W. C. Ko, and *Homalium tomentosum* (Vent.) Benth. (Gagnepain 1921, Lescot 1970, Hô 1999, Yang and Zmarzty 2007). Here, we describe an additional species, *Homalium glandulosum* Tagane & V. H. Nguyen, from Vu Quang National Park, Ha Thinh Province, North Central Vietnam.

**Figure 1.**
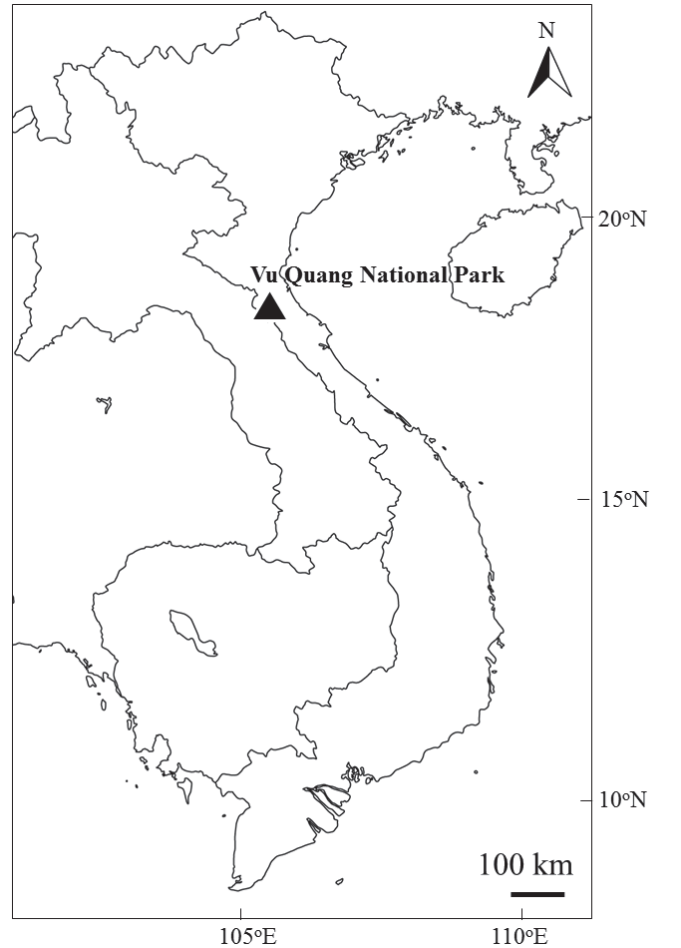
Location of Vu Quang National Park, Vietnam.

Vu Quang National Park covers an area of ca. 56,000 ha containing an elevation gradient of over 2,000 m, from 30 m in the lowlands to 2,286 m at the summit of Mt. Rao Co (Rào CÓ), on the border with Laos (Vu Quang National Park Management Board 2014: see Fig. 1). The vegetation is diverse along the elevational gradient and Kuznetsov (2001) described five major forest types: lowland forests (alt. 10–300 m), hill forest (alt. 300–1,000 m), medium montane forest (alt. 1,000–1,400 m), montane forest (alt. 1,400–1,900 m) and upper montane forest (alt. 1,900–2,100 m). From the national park, 1,678 species of vascular plants, 94 species of mammals, 315 species of birds, 58 species of reptiles and 31 species of amphibians, including many endemic and rare species, have been recorded, indicating that Vu Quang National Park is one of the centers of biodiversity in Vietnam (Eames et al. 2001, Tordoff et al. 2004, Vu Quang National Park Management Board 2014).

During our botanical inventory in Vu Quang National Park in July 2015, we discovered a previously undescribed species of the genus *Homalium*. Here we describe the species as *Homalium glandulosum*, accompanied with illustrations, DNA barcodes of the two plastid regions *rbcL* and *matK* (CBOL Plant Working Group 2009), and a key to the species of *Homalium* in Vietnam. DNA amplification and sequencing were performed according to published protocols (Kress et al. 2009, Dunning and Savolainen 2010, Toyama et al. 2015).

## Taxonomy

### Homalium glandulosum

Taxon classificationPlantaeMalpighialesSalicaceae

Tagane & V. H. Nguyen sp. nov.

urn:lsid:ipni.org:names:77151885-1

Figures 2 , 3

#### Diagnosis.

Similar to *Homalium petelotii* Merr., but differing in having distinct glands, often stalked, at base of lamina and along margin of stipules and bracteoles, and spreading hairs on rachis of inflorescences, calyx tubes, sepals and petals (vs. glabrescent or only short appressed hairs in *Homalium petelotii*). Also, similar to *Homalium cochinchinense* (Lour.) Druce and *Homalium mollissimum* Merr. but distinguished from these two by the distinct glands mentioned above and very sparsely pubescent branches and petioles (vs. pubescent to densely pubescent).

#### Type.

VIETNAM. Ha Tinh Province; Vu Quang National Park; along the trail to the summit; in hill forest, alt. 453 m, 18°16'25.3"N, 105°21'40.8"E, 25 July 2015, *Tagane S., Yahara T., Toyama H., Nguyen N., Yang C. J. & Nguyen H. V3735* (holotype KYO!; isotypes BKF!, DLU!, FU!, NTU!, the herbarium of Vu Quang National Park!).

#### Description.

Small tree, 9 m tall, DBH 15.6 cm; bark gray-brown; branchlets very sparsely pubescent, soon glabrous, with many lenticels, which are narrow to broadly elliptic, 0.3–0.9 × 0.1–0.25 mm, whitish; young twigs blackish when dry, old twigs grey-brown. Leaves: simple, alternate, petiole 2.5–5 mm long, sparsely pubescent when young, blackish when dry; leaf blade ovate to elliptic-ovate or oblong-ovate, (2.4–)3.5–9.8 × (1.0–)1.2–3.1 cm, papery, very sparsely pubescent on both surfaces, apex acuminate to acute, rarely obtuse, with a gland on tip, base cuneate, with 2–5 pairs of stalked glands at border with petiole, margin crenulate with glandular teeth; midribs prominent on both surfaces, glabrescent, secondary veins 6–7 pairs, arising at an angle of 40–45 degrees from the midrib, slightly prominent on both surfaces, tertiary veins reticulated, visible when dry. Stipules narrowly triangular, ca. 5 × 1 mm, thinly papery, with 4–6 glandular teeth per side, glands often stalked, apex acute, with a gland on tip, blackish when dry, very sparsely pubescent, caducous. Inflorescences axillary, pendant, racemes or racemiform panicles with flowers borne singly on rachis or in clusters of up to 3 sometimes on short branches, 4–9 cm long, 7–20 flowered; rachis densely pubescent with spreading hairs except glabrous and lenticellate basally; bracts caducous, not seen. Pedicels 2.5–3.5 mm long, densely pubescent with spreading hairs; bracteoles narrowly ovate, 2.5–3 mm long, margin with 3–5 stalked glands per side, glabrous except near base, caducous. Flowers fragrant, 6–8 mm in diam.; calyx tube ca. 2.5 mm long, densely pubescent with spreading hairs; sepals 8, narrowly oblanceolate, ca. 3 × 0.5 mm long, membranous, with conspicuous midvein, light green *in vivo*, glabrescent except near basal part on both surfaces and ciliate margin, hairs spreading, 0.6 mm long, apex apiculate, with a gland on tip; petals 8, oblanceolate, 3.5 × 1.1 mm, membranous, with conspicuous midvein and lateral veins, whitish *in vivo*, glabrescent except lower 1/3 on both surfaces, margin ciliate with spreading hairs, hairs ca. 0.6 mm long, apex obtuse to acute. Disk glands 8, ca. 0.3 mm in diam., orange *in vivo*, black when dry, stipitate, stalk ca. 0.15 mm long, sparsely hairy. Stamens 8, filaments ca. 5.5 mm long, sparsely spreading hairy in lower 3/5; anthers ca. 0.4 mm long, longitudinally and extrorsely dehiscent. Ovary semi-inferior. Styles 3 or 4, filiform, ca. 5 mm long, united in lower 1/3, hairy in lower 1/2, hairs spreading; placentas 3 or 4, each with (3–)4 ovules, sparsely hairy inside. Fruits not seen.

**Figure 2.**
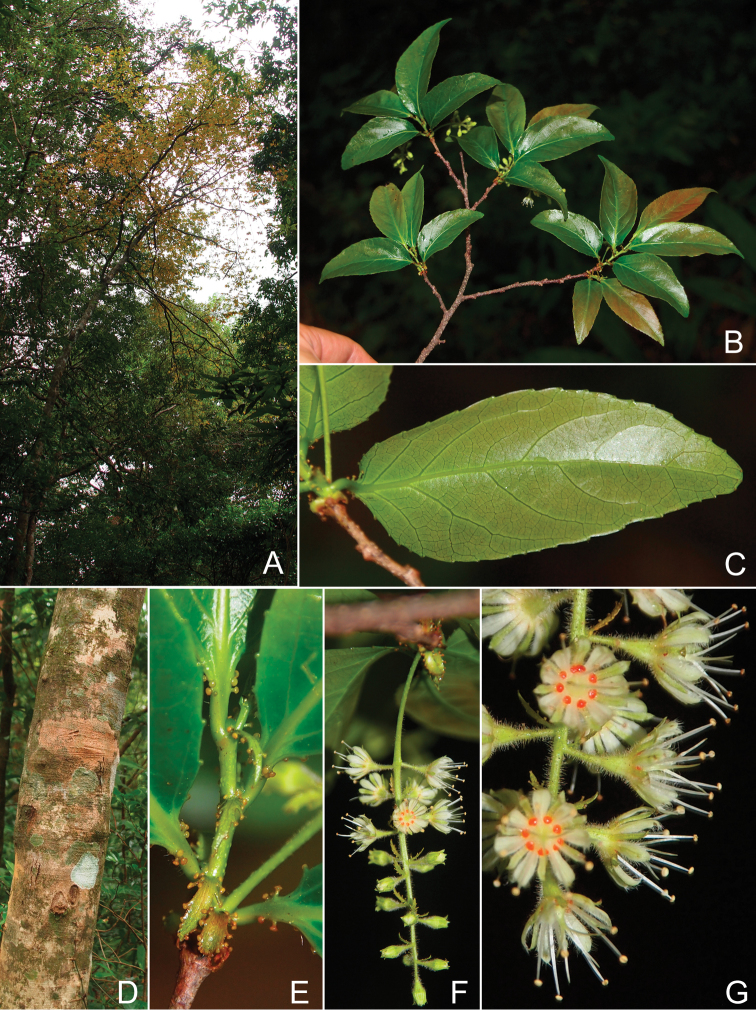
*Homalium glandulosum* Tagane & V. H. Nguyen: **A** habit **B** a flowering branch **C** abaxial leaf surface **D** bark **E** apical branch showing glandular stipules and leaf base **F** inflorescence **G** close up view of flowers.

**Figure 3.**
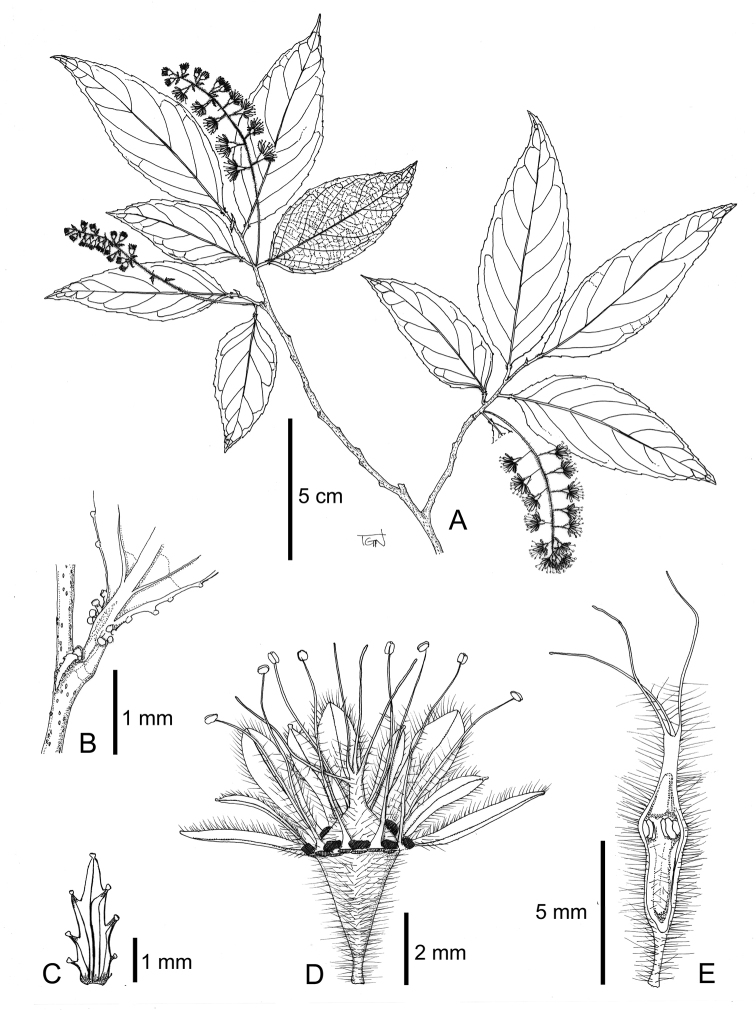
*Homalium glandulosum* Tagane & V. H. Nguyen: **A** flowering branch **B** stipule and base of a leaf **C** bracteole **D** flower **E** longitudinal section of gynoecium.

#### Distribution.

So far known only from the type locality.

#### Habitat and Ecology.

Rare in hill evergreen forest, at alt. 453 m. Flowering specimens were collected in July.

#### GenBank accession no.

*Tagane et al. V3735*: LC0901208 (*rbcL*), LC0901207 (*matK*). The BLAST similarity search based on the *matK* sequence of *Homalium glandulosum* resulted in homology as high as 834/835, 773/774, 767/768 bp with the sequence of *Homalium cochinchinense* (GenBank accession no. HQ415362, KP093841, KP093840, respectively) in the DNA database.

#### Etymology.

The specific epithet ‘*glandulosum*’ reflects the existence of distinct glands, often stalked, on stipule, leaf base and bracts.

#### Conservation status.

Data Deficient. Only one flowering individual was found in a protected area of Vu Quang National Park. Further efforts for finding additional individuals/populations are needed to evaluate its conservation status.

#### Note.

In Vu Quang National Park, another species of *Homalium*, *Homalium cochinchinense*, occurs in lowland forest (e.g., alt. 70 m, 27 July 2015, *Tagane et al. V3818* (BKF!, DLU!, FU!, NTU!, the herbarium of Vu Quang National Park!). The species is easily distinguished as in the above diagnosis and the following key.

### Key to the species of *Homalium* in Vietnam (modified from Lescot (1970), Sleumer (1985) and Yang and Zmarzty (2007))

**Table:** 

1a	Stamens solitary before each petal	**2**
1b	Stamens 2 or more before each petal	**8**
2a	Styles dentiform, less than 1 mm long	***Homalium tomentosum***
2b	Styles filiform, 2–5 mm long	**3**
3a	Petals less than 2 mm long	***Homalium ceylanicum***
3b	Petals 3–4 mm long	**4**
4a	Stipules, leaf bases and bracts with stalked glands	***Homalium glandulosum***
4b	Stipules, leaf bases and bracts without stalked glands	**5**
5a	Petioles 8–15 mm long; leaves with acumen ca. 10 mm or more; leaf blade drying blackish brown	***Homalium phanerophlebium***
5b	Petioles less than 7 mm long; leaves with acumen 9 mm or less; leaf blade not drying blackish (i.e. reddish brown to dark greyish brown)	**6**
6a	Inflorescences glabrescent or pubescent only with short appressed hairs	***Homalium petelotii***
6b	Inflorescences pubescent with spreading trichomes	**7**
7a	Abaxial surface of leaf pubescent on midrib and lateral veins only	***Homalium cochinchinense***
7b	Abaxial surface of leaf sparsely to densely pubescent throughout	***Homalium mollissimum***
8a	Stamens partly inserted on the lower part of the petals; sepals manifestly accrescent after anthesis	**9**
8b	Stamens inserted strictly between the disk glands; sepals not or slightly accrescent after anthesis	**10**
9a	Bracts lanceolate-oblong, 4–8 mm long, caducous	***Homalium grandiflorum***
9b	Bracts ovate-flabelliform, 5–6 mm long, persistent	***Homalium dictyoneurum***
10a	Stamens consistently 2 before each petal	***Homalium dasyanthum***
10b	Stamens at least partly in fascicles of 3 or more	**11**
11a	Flowers with distinct pedicles of 3–5 mm long; calyx tube same length as the petals	***Homalium myriandrum***
11b	Flowers subsessile; calyx tube more than twice as long as petals	***Homalium caryophyllaceum***

## Supplementary Material

XML Treatment for Homalium glandulosum

## References

[B1] Applequist WL (2013) A nomenclator for *Homalium* (Salicaceae). Skvortsovia 1(1): 12–74.

[B2] CBOL Plant Working Group (2009) A DNA barcode for land plants. Proceedings of the National Academy of Sciences of the United States of America 106: 12794–12797. doi: 10.1073/pnas.0905845106 19666622 10.1073/pnas.0905845106 PMC2722355

[B3] Chase MW Zmarzty S Lledó MD Wurdack KJ Swensen SM Fay MF (2002) When in doubt, put it in Flacourtiaceae: a molecular phylogenetic analysis based on plastid *rbcL* DNA sequences. Kew Bulletin 57: 141–181. doi: 10.2307/4110825

[B4] Dunning LT Savolainen V (2010) Broad-scale amplification of *matK* for DNA barcoding plants, a technical note. Botanical Journal of the Linnean Society 164: 1–9. doi: 10.1111/j.1095-8339.2010.01071.x

[B5] Eames JC Eve R Tordoff AW (2001) The importance of Vu Quang Nature Reserve, Vietnam, for bird conservation, in the context of the Annamese Lowlands Endemic Bird Area. Bird Conservation International 11: 247–285 doi: 10.1017/S0959270901000326

[B6] Gagnepain F (1921) Homaliacées. In: Lecomte MH Gagnepain F (Eds) Flore générale de l’Indo-Chine 2 Masson, Paris, 1005–1015.

[B7] Hô PH (1999) Cay Co Viet Nam: An Illustrated Flora of Vietnam Vol. 1, Montreal.

[B8] Kress WJ Erickson DL Jones FA Swenson NG Perez R Sanjur O Bermingham E (2009) Plant DNA barcodes and a community phylogeny of a tropical forest dynamics plot in Panama. Proceedings of the National Academy of Sciences of the United States of America 106(44): 18621–18626. doi: 10.1073/pnas.0909820106 19841276 10.1073/pnas.0909820106 PMC2763884

[B9] Kuznetsov A (2001) The forests of Vu Quang Nature Reserve: a description of habitats and plant communities. WWF, Hanoi.

[B10] Lescot M (1970) Flacourtiaceae. Flore du Cambodia, Laos du Vietnam 11: 3–98.

[B11] Sleumer H (1954) Flacourtiaceae. Flora Malesiana (ser. I) 5: 1–106.

[B12] Sleumer H (1973) Révision du genre *Homalium* Jacq. (Flacourtiacées) en Afrique (y compris Madagascar et les Mascareignes). Bulletin du Jardin Botanique National de Belgique 43: 239–328. doi: 10.2307/3667612

[B13] Sleumer H (1985) The Flacourtiaceae of Thailand. Blumea 30: 217–250.

[B14] Tordoff AW Tran QB Nguyen DT Le MH (2004) Sourcebook of existing and proposed protected areas in Vietnam. Birdlife International in Indochina and Ministry of Agriculture and Rural Development, Hanoi.

[B15] Toyama H Kajisa T Tagane S Mase K Chhang P Samreth V Ma V Sokh H Ichihasi R Onoda Y Mizoue N Yahara T (2015) Effects of logging and recruitment on community phylogenetic structure in 32 permanent forest plots of Kampong Thom, Cambodia. Philosophical Transactions of the Royal Society B: Biological Sciences 370(1662): . 10.1098/rstb.2014.0008 PMC4290422 25561669

[B16] Vu Quang National Park Management Board (2014) Planning for conservation and development of Vu Quang National Park 2015–2020. [In Vietnamese; published by author]

[B17] Yang Q Zmarzty S (2007) Flacourtiaceae. In: Zhengyi W Raven PH Deyuan H (Eds) Flora of China 13: 112–137. http://www.efloras.org

